# Supplementary material for: Are carbon emissions trading and green financial instruments synergistic? -Comprehensive quantitative research based on content analysis
Source: PLoS One. 2024 Mar 7;19(3):e0298601. doi: 10.1371/journal.pone.0298601 (PMC10919601; doi:10.1371/journal.pone.0298601)
Supplement: S1 Data — (ZIP) [file pone.0298601.s001.zip › Data/地级市Z_F工作报告.zip!/░▓╗╒/┬φ░░╔╜╩╨/└┤╫╘╣1⁄2╓┌║┼í╛┬φ┐╦╩2╛▌═°í┐_2003.docx]

各位代表：

现在，我代表市第十二届人民政府，向大会作政府工作报告，请予审议，并请市政协委员和其他列席人员提出意见。

一、2002年和过去五年工作的回顾

2002年，在省委省政府和市委领导下，市政府团结和依靠全市人民，坚持以经济建设为中心，全面加大各项工作推进力度，较好地完成了市十二届人大五次会议确定的任务，全市经济和社会发展取得了新成绩。

(一)经济发展全面提速增效。全年完成国内生产总值155亿元（预计，下同），比上年增长12.7％，超预期增幅3.7个百分点，表明我市经济经过结构调整步入了稳健攀升的轨道。工业经济增长速度和效益全面提升，全部工业总产值153亿元，比上年增长22％；规模以上工业企业销售收入183亿元，增长23％；综合经济效益指数113，提高16个百分点。马钢生产经营和经济效益再创新水平，在全市经济增长中继续发挥主力作用。市属工业在星马、山鹰等骨干企业和一批外来投资企业的强力拉动下增势强劲，县区工业呈现加速兴起势头，市属、县区工业对全市工业增长的贡献率达70％。农业在结构调整中稳定发展，总产值10.5亿元，比上年增长3％。乡镇企业回升加快，营业收入70.6亿元，比上年增长8.6％；实现税收1.12亿元，增长16％。商贸流通业通过改制重组和开放引进，发展活力明显增强，完成社会消费品零售总额41.3亿元，比上年增长8％。苏果超市销售额再增三成。天润发、上海联华、华联、农工商超市集团接连进入，浙江海外海汽车贸易城的开工建设，本地一批流通、餐饮企业的扩张，推动全市商贸流通业进入整合和提升的发展阶段。采石风景区被批准为国家级风景名胜区，强化了濮塘风景区管理，旅游业得到新的发展。个体私营经济增加值32.4亿元，比上年增长19％，占国内生产总值比重上升到21％；实现税收1.3亿元，增长30％。各领域的固定资产投资高速增长，形成新一轮投入高峰，预计全社会固定资产投资58亿元，比上年增长66.9％；投资结构更加合理，技改投资增长91.8％。在经济发展和征管加强的基础上，各项税收大幅度增长，市、县、区财政全面超收，全市财政收入23.14亿元，比上年增长16％，超预期增幅７个百分点；财政支出结构得到调整和优化。金融机构加强风险监管，积极支持经济建设，年末存贷款余额分别比年初增加21.5亿元和4.7亿元。

(二)对外开放取得明显突破。开发区和县区工业、旅游园区建设步伐全面加快，开放型经济的优良载体正在加速构建。开发区西区路网骨架基本形成，提前实现了3.2平方公里的开发目标，东区建设进入启动阶段，并已开始规划跨过采石河向南推进。当年入驻项目27个，工业产值达到15亿元，开发区聚集投资、促进增长的作用初步显现。慈湖工业园和三个区工业、旅游园规划建设全面展开，当涂工业园框架初步拉开，招商工作取得一定进展。通过年初解放思想大讨论和招商引资目标责任制的推行，全市“敞开东大门，实现大开放”的浓厚氛围开始形成，各领域全面开放、全员招商的新局面初步显现。日本大同、台湾中橡等企业增资扩股，北京首创、广东格力、浙江万马等国内著名企业进入我市投资兴业。全年实际利用外资4300万美元，是上年的2.4倍；利用省外资金８亿元，比上年增长一倍。对外贸易继续发展，进出口总额3.3亿美元，比上年增长15.1％。马钢积极应诉美国Ｈ型钢反倾销调查并取得胜诉。对外工程承包、劳务合作、设计咨询等取得新进展。与国际友城的合作深入拓展，和西班牙阿尔冈达·德雷伊市结为友好城市。

(三)以国企改革为中心的体制改革深入推进。以“政府置换土地、企业置换产权、职工置换身份、健全社会保障”为主要内容的产权制度改革，在工、商、交、建企业全面实施，取得了突破性进展，已有24户大中型企业进行了产权多元化的改制。马钢、十七冶等企业的学校剥离工作正在加紧准备，即将实施。矿院、设计院转制进入市场的活力进一步显现。全年1.1万余名国有企业下岗职工顺利出中心，提前一年基本实现全市国企下岗职工由基本生活保障向失业保险转轨并线的目标，为国有企业改制和资产重组创造了条件。以明确投资和经营主体，强化国有资产保值增值为主要目的，建立了国有资产管理运营新体制框架。对城区管理体制实施了重大改革，将６个方面的36项事权和165户国有集体中小企业下放到三个区，同时完善市与区财政体制，全面塑造经济和社会发展的权力、责任、利益主??险覆盖面扩大、征缴率提高，克服困难将矿院、设计院养老保险纳入全市统筹。对城镇职工基本医疗保险政策进行了重大调整和完善，覆盖面扩大到22.6万人，减轻了广大职工医疗费用负担。城市居民最低生活保障面达到1.6万户3.6万人，基本实现了“应保尽保”。城市户籍制度改革取得良好效果，已有2.2万人办理了进城落户手续。经过新一轮市县机构改革，机构、编制分别精简26％和30％，分流人员得到妥善安置。

(四)城乡基础设施建设步伐进一步加快。围绕城市发展新目标，启动了新一轮城市总体规划修编，完成了新区控制性规划和部分专业规划。在规划指导下，城市“东扩南进”框架迅速拉开。江东大道南北延伸段、红旗南路中段拓宽如期完成，湖西南路、九华路等骨干道路提前竣工，313省道三期工程、马濮旅游公路加紧建设。公用设施配套建设全面加快。雨山湖分流工程全线竣工。第四水厂、第二污水处理厂、天然气利用工程动工兴建。房地产开发通过整顿、规范，开始步入健康发展的轨道，全年房地产投资额达10亿元，比上年增长31.2％；开工面积80万平方米，竣工面积54万平方米，分别比上年增长１７％和４％。土地市场进一步依法规范，国有土地资本运营取得显著效果。小城镇建设取得新进展，当涂县城和一批重点镇的服务功能得到增强。国债水利项目高质量实施，长江马鞍山河段整治一期工程、重要堤防除险加固和一批排涝泵站建设项目顺利完成，沿江山丘区抗旱工程扎实推进，提高了防洪排涝抗旱能力。为了建设适宜创业和居住的环境，全面启动创建国家环保模范城工作。

(五)人民生活水平继续提高。把就业作为民生之本，采取多种措施促进就业和再就业，全年新增就业岗位1.6万个，城乡劳务输出8.4万人，城镇登记失业率为4.2％。各级财政增加社会保障和困难救助的支出，确保了下岗职工基本生活费、离退休人员养老金和城市居民最低生活保障金的按时足额发放。通过发展经济、扩大就业、各种要素参与分配，居民收入增速加快，城镇居民人均可支配收入和农民人均纯收入分别比上年增长10.5％和６％，超预期目标5.5和３个百分点。年末城乡居民储蓄存款比年初增加14.3亿元。城市人均住房建筑面积比上年增加0.8平方米；物业管理进一步规范。安排到我市的350名三峡移民得到妥善安置。年初安排的老旧小区和背街小巷整治一期工程、农村饮用水工程、“信息入村”等８件为民办实事项目，已完成６件，２件按计划实施。

(六)科技教育和各项社会事业取得新进展。高新技术创业服务中心经过一年紧张建设投入使用，入驻企业１８家，开始发挥科技产业“孵化器”作用。矿院国家级金属矿山固体废物处理与处置工程技术研究中心获得批准。我市再次获得“全国科技进步先进市”称号。中小学危房改造和布局调整取得成效。发展民办教育取得新突破。安工大瞄准目标不懈努力，各方面给予通力支持，进入快速发展轨道，东校区一期工程顺利建成，在校生规模扩展到1.5万人，软件职业技术学院正在积极筹建之中。高等师范专科学校加紧筹建，新校区一期主体工程基本竣工。利用国内著名高校资源创办的网络大学开始投入建设。群众性文化活动和专业文艺创作活跃。成功发行一枚采石矶特色邮资信封。广播电视服务质量进一步提高。疾病预防措施得到积极落实。竞技体育在省十运会上取得好成绩。人口出生率控制在省下达的13‰以内。文明创建活动多层次深入开展，创建文明城市工作实现比较稳定的良性循环。

一年来，依法行政工作扎实推进。市政府承办的148件人大代表议案、建议和307件政协提案按要求办复。着力于整治经济发展环境、转变政府职能，全面清理全市各类政策规定，宣布废止和失效179件；改革行政审批制度，取消了106项行政审批事项，取消降低了18项收费。进一步完善市、县行政服务中心运作机制，努力为投资者和广大群众提供有效服务。以成品油、集贸、旅游、房地产四个市场为重点的各类市场秩序整治取得阶段性成果。安全生产基础工作继续全面强化，重大安全隐患得到果断整治。行政监察工作深入推进，廉政建设得到加强。“四五”普法深入开展。社会治安形势保持稳定。国防动员、民兵预备役、“双拥”和人防工作继续加强。各级信访组织为化解社会矛盾作出了积极贡献。其他各项工作都有新的进步。

2002年取得的令人鼓舞的成绩，标志着市十二届人大一次会议确定的五年主要任务已经顺利完成。过去的五年，是跨越世纪的五年，是马鞍山经受体制转轨、城市转型的阵痛，经受亚洲金融危机影响和市场急剧变化的冲击，经受连续两年长江特大洪水袭击的严峻考验，战胜困难扎实前进、取得显著成就的五年。

这五年，综合经济实力显著增强。五年间，国内生产总值年均增长9.6％，人均国内生产总值突破万元，2002年达1.3万元；工业总产值年均增长11.3％；农业总产值年均增长3.5％；社会消费品零售总额年均增长7.2％；外贸进出口总额年均增长7.9％；财政收入五年累计96.7亿元，年均增长9.4％；固定资产投资逐年加大，累计完成181.4亿元，年均增长10.2％，其中国债资金８亿元，一批骨干项目相继建成，促进了即期增长，增强了发展后劲。

这五年，结构调整取得重大进展。工业结构发生了积极变化，支撑经济增长的能力不断增强。马钢通过技术改造，产品竞争力明显提升。市属工业通过改制、改造和引进，骨干企业群体加速形成。马钢年销售收入在全省率先突破百亿元，星马、山鹰公司分别突破１０亿元、５亿元，销售收入过亿元企业增加到１３家，由马钢和一批骨干企业领跑的工业梯次发展格局开始形成，规模以上工业增加值占全市工业比重提升到８５％。农业结构得到调整优化，主要农产品优质品率达９０％以上，经济作物占农作物比重达５０％，养殖业产值比重提升到６０％。民营经济的持续扩张和外来资本的不断进入，带动了所有制结构和产业结构的调整。个体私营经济税收年均增长１２％，梦都、翠林等在竞争中崛起的品牌企业已走出城门，向外拓展连锁经营。非公有制经济占全市经济的比重达到35％。一、二、三次产业比重由1997年的12：61.8：26.2调整为8.5：59.9：31.6。

这五年，改革开放向纵深推进。市场化取向的改革力度不断加大，市场经济体制框架基本形成。国有企业改革攻坚克难，向纵深发展。马钢内部改革、主辅分离取得新进展。十七冶在开拓市场中开始显现生机。市属企业进入资本市场取得突破，山鹰公司成功上市并获准增发可转换债券，星马公司上市已获批准，天源科技等企业进入上市辅导期。天成纺织等近百户国有集体企业实施了改制，催生和壮大了一批新的增长点，激活了一大批存量资产。乡镇企业改制面达到９９％。土地二轮承包改革顺利完成。农村税费改革稳步推进，减轻了农民负担。住房制度改革不断深化，新的住房体制基本建立。以养老、失业、医疗保险和城市居民最低生活保障为主要内容的社会保障体系基本形成，农村社保体系建设开始启动。市区行政区划调整、社区组建、市与区的事权财权划分、相对集中行政处罚权试点、新一轮市县机构改革等工作顺利实施，行政管理体制改革跨出了重要的一步。以启动开发区建设为重点，不断加大对外开放力度，五年累计实际利用外资1.3亿美元、内资２４亿元。对外经济合作、友好交流都有新的发展。

这五年，城乡面貌发生新的变化。完成了３１４省道改建，马芜高速公路、３１３省道等一批项目加快建设，对外交通网络正在加速形成。新建、改建、扩建３０多条主次干道，初步形成了“七纵七横二环一线”的城市道路网络，正在加紧拉开百万级人口城市的构架。金家庄旧城改造取得显著成效。公用设施建设快速推进，承载、服务功能得到增强。小城镇建设取得明显进展。城市环境综合整治定量考核连续五年名列全省前茅。五年来，在巩固国家卫生城市、园林城市成果的基础上，先后荣获中国优秀旅游城市、全国创建文明城市工作先进市称号，以及中国人居环境范例奖、迪拜国际改善居住环境良好范例奖。

这五年，人民生活得到较大改善。五年间，城镇居民人均可支配收入由６１０８元增加到７６５０元，农民人均纯收入由２５４５元增加到２９３０元。城市人均住房建筑面积由１７．５平方米增加到２１平方米，物业管理覆盖面扩大到８５％；农村人均住房面积由２５平方米增加到３２平方米，居住质量得到提高。五年中，近２万名下岗职工实现了再就业，越来越多的农民进入城镇务工、创业和生活。城乡困难群众的生活得到基本保障。市政府实施６９件为民办实事项目，解决了一批群众关注的热点、难点问题。

回顾五年来的实践，我们深深地感到，马鞍山作为一个受传统体制影响较深、以传统产业为主体的工矿城市，要走上一条符合时代要求、具有自身特点的加快发展之路，第一，必须摒弃一切不合时宜的思想观念，坚决突破思想观念上的种种障碍，在实践中解放思想，在解放思想中统一思想，敢为人先，勇于创新，放胆迈步闯出发展新路；第二，必须保持奋发有为的精神状态，以宽广的视野审视自己，以更高的目标鞭策自己，敢于和先进地区比拼，攻坚克难，奋力拼搏，不断开拓前进；第三，必须把发展作为第一要务，以加快发展为主题，集中力量于经济建设主战场，抢抓机遇发展经济，以加快发展促进结构调整，以加快发展解决前进中的困难和问题；第四，必须把开放型经济作为最具带动力的经济龙头，以招商引资为重中之重，强力实施大开放主战略，全面推进体制创新、科技创新，为生产力的发展注入不竭的动力；第五，必须把提高人民生活水平作为出发点和归宿，正确处理改革、发展、稳定的关系，最大限度地调动和发挥一切积极性、创造性，凝聚各方面力量，万众一心奔向富民强市的宏伟目标。

过去五年的成就确实来之不易，这是认真贯彻中央、省委省政府的决策部署，在市委正确领导下，在市人大市政协监督支持下，全市人民团结一心、不畏艰难、负重奋进、共同努力的结果。在此，我谨代表市第十二届人民政府，向全市广大工人、农民、知识分子和各级干部，向各民主党派和无党派爱国人士、各群众团体，向国家和省驻马单位、解放军驻马部队、武警官兵，向所有关心支持马鞍山发展的同志们和朋友们，表示衷心的感谢！

总结过去的五年，我们也清醒地看到，马鞍山在发展中还面临不少困难和矛盾，政府工作中还存在许多问题和不足，主要表现在：一是思想观念、行为方式不适应大开放的要求，开放型经济滞后对经济、社会发展的影响广泛而深刻。二是体制机制不适应发展市场经济的要求，改革还面临十分艰巨的攻坚任务。三是经济结构不合理的矛盾仍然突出，产品科技含量不高、传统产业竞争力不强、新兴产业发展缓慢、个体私营经济和县区经济实力比较薄弱。四是城镇就业不足和农民增收不快的矛盾仍然突出，困难群体还需要从多方面进行救助。五是政府系统一些干部的观念、本领和作风与加快发展的要求、人民群众的期望还有较大差距，官僚主义、形式主义在各级政府机关中还不同程度地存在，纠正部门和行业不正之风、加强廉政建设的措施还需要进一步落实。对这些困难和问题，需要进一步采取有效措施，切实加以解决。

二、今后五年的奋斗目标和主要任务

新世纪的前二十年，是按照党的十六大的战略部署，全面建设小康社会的新的历史阶段，是重要的战略机遇期。今后五年，是这一发展新阶段中至关重要的起步期，是马鞍山改革攻坚期、发展提速期、向更高目标奋进的跨越期。

当前，国际资本向我国加速流动，沿海资本向内地加速转移，以上海为核心的长江三角洲加速崛起，皖江开发开放加速拓展，为我市发挥产业基础和区位条件，更加有效地利用外来投资加快结构调整、加快生产力发展提供了更多的现实机遇。同时，随着经济全球化、市场化的快速推进，沿海发达地区及周边城市在高起点上加快发展，也使我市面对着更大的竞争压力。我们必须审时度势，以强烈的危机感和使命感，以拼搏进取的昂扬姿态抢抓机遇、迎接挑战，全力以赴把我市现代化建设推向加速发展的新阶段。今后五年，要在全面完成“十五”计划的基础上，到２００７年国内生产总值比２０００年翻一番，实现物质文明、政治文明和精神文明共同进步，为提前实现国内生产总值翻两番、全面建设小康社会奠定坚实的基础。主要预期目标为：国内生产总值年均增长１２％；财政收入基本同步增长；固定资产投资年均增长２０％；城镇居民人均可支配收入和农民人均纯收入年均分别增长８％和５％，城镇登记失业率控制在４．５％以内。确定这样的目标，是在加快皖江开发开放中率先突破的内在要求，是加快发展、富民强市的必然选择，通过努力也是完全有可能实现的。我们要奋力拼搏，力争完成得更好一些。

今后五年的主要任务是：

——对外开放取得新突破。加大力度实施大开放主战略，全方位、多层次、宽领域扩大对外开放，构建加快发展的新平台。五年力争实际利用外资５亿美元、内资１００亿元，年均增长３５％；进出口总额保持两位数增幅。到２００７年，外来投资占固定资产投资达２０％以上，进出口总额占国内生产总值的比例达２５％。

大力构建以开发区为龙头的一区五园体系。五年内完成各类园区开发面积３０平方公里，整体塑造形象，形成规模产出能力，使园区成为经济增长和技术创新的基地。开发区西区要形成较大的产出规模，全面建成东区，跨过采石河发展南区，力争完成开发总面积１５平方公里以上。慈湖工业园完成基础设施配套，支持现有企业增资扩股，并力争有一批骨干工业项目入驻，形成大中型骨干企业集中区。县区工业、旅游园基本完成功能开发，招商引资取得重要进展，成为县区经济强劲增长的动力源，带动县区经济跻身全省先进行列。同时，支持一批特色乡镇工业园的规划建设。

以招商引资为重点推动开放型经济加快发展。深入发动，强化责任制，形成全民招商、全市引资的局面。以工业为重点，以园区为载体，着力引入国际跨国公司、国内上市公司，以及沿海地区特别是浙江、广东的民营企业。突出支持马钢、星马、山鹰等大企业和大单位招商引资，把骨干企业推向招商的前沿，把优质资产和好项目拿出来招商，以大引大，以优引优，以强引强，以增量做大存量，形成优质要素聚集效应。通过不懈的努力，大幅度提高外来投资企业在经济总量、税收、固定资产投资中的比重，加速形成公有制经济、外资经济、民营经济合力驱动经济增长的格局。以外资促外贸，培育新的出口创汇主体和基地，让更多的企业、产品进入国际市场，形成比较稳定的出口增长力量。积极实施“走出去”战略，支持有条件的企业进入国际市场开拓发展空间。

以更加积极的姿态加速融入长江三角洲。主动接受长江三角洲的辐射，尤其要利用上海的资源和“申博”成功带来的商机，借势发力，错位发展，形成比较优势，使马鞍山逐步成为与长江三角洲互动发展的现代加工制造业基地、绿色食品供应基地和富有特色的休闲旅游基地。全面参与南京都市圈建设，尽快在交通等基础设施对接上取得更大进展，在产业融合上收到明显成效。寻求与江宁区合作，突破行政区划共谋发展。坚持开发开放双向促进，积极与皖江地区尤其是芜湖、铜陵加强协作，合力打造“马芜铜”城市群品牌，努力在加快皖江开发开放中率先突破。

坚持不懈地整治和改善发展环境。牢固确立环境是发展第一要素的意识，坚持从强化责任抓起，从解决突出问题抓起，从法制和制度建设抓起，不断优化政策、信用、人文等环境，使马鞍山投资创业环境更具吸引力和竞争力。

——结构调整形成新格局。坚持以结构调整为主线，在发展中推动结构调整，在调整中实现加快发展，为全面建设小康社会奠定坚实的物质基础。

走新型工业化之路建设工业强市。培育多元化投入主体，尤其要全力引进加工制造业项目，以信息化带动工业化，加速构建新型工业化体系。支持马钢加快技术改造，并运用有效资源发展深加工和非钢产业，使非钢产业比重有明显提高，实施低成本扩张、高效率发展。市属、县区工业要通过市场化运作和必要的扶持，着力推进骨干项目建设，主攻专用汽车及汽车零部件、新材料、精细化工、生物医药、造纸、磁性材料等支柱产业和高新技术产业，推动骨干企业规模化、中小企业优质化，形成大而强、专而精的企业群体，使马鞍山由产品结构比较单一的重工业基地，加快向形成一批支柱产业的现代加工制造业基地跨越。到２００５年，马钢销售收入要达１５０亿元，２００７年力争再上一个新台阶，继续走在全省工业企业前列；到２００７年，市属和县区工业争取有１户企业销售收入超５０亿元，２－３户企业超１０亿元，５户企业超５亿元，２０户企业超亿元，规模以上工业企业超２００户，力争规模以上工业企业销售收入年均增长２０％以上，牵动全市经济快速发展。

围绕农民增收加快农业和农村经济结构的战略性调整。以工业理念和措施调整农业结构，发展农村经济。在推动现有产业化龙头企业加速发展的同时，积极利用外来资本、民间资本、工商资本培育和发展加工型龙头企业，大力发展“订单农业”。到２００７年，争取引进和培育出销售收入超亿元的龙头企业５户以上，带动更多的农户增收。坚持城郊型农业的结构调整取向，围绕市场抓农业，实施农业综合开发，建立健全农产品质量标准体系、检验检测体系和认证体系，积极发展绿色食品、有机食品和无公害农产品，建设一批特色鲜明、规模效益突出的生产基地，把重点面向长江三角洲城市居民消费的“餐桌经济”做大做强，并力争在农产品出口上取得突破。以培育块状经济、配套经济、特色经济、劳务经济为着力点，以发展个体私营经济为切入点，促进乡镇企业的振兴。全力推广农业科技，通过有组织的培训，提高农民科技文化素质，使全市良种良法逐步实现全面覆盖，使农业技术水平和劳动生产率有较大幅度的提高。利用世贸组织有关规则，合理有效地加强对农业的扶持，促进农业增效、农民增收、农村稳定。

大力推进现代服务业的加快发展和传统服务业的全面振兴。积极创造条件，加速服务业的对内对外开放，开发就业经济、税源经济，促进产业协调发展。继续引进有实力的企业，借助外力与民间资本加快商贸流通等传统产业的整合和改造，基本完成企业重组和业态调整。依托区位条件，积极发展物流配送产业。把旅游业、房地产业作为潜力产业加以精心培育。实施采石古镇及周边的整体开发，打响采石风景区品牌，形成旅游业龙头板块。以花山旅游园规划建设为载体，加快城东开发和濮塘风景区的建设，构造旅游业集散功能，形成服务业发展新亮点。推进雨山湖、青山、横山风景区建设，营建具有特色的景区景点体系。引导房地产企业进行规模化开发、集约化营销，全面加强物业管理，提升开发质量，满足市民对住房的多层次需求，增强对周边居民的吸引力，带动全市房地产业健康快速发展。积极培育和引进各类中介机构，加快发展社区服务业，促进第三产业整体繁荣。

增强结构调整的支撑力量。加强全市科技资源的整合，建立有效的科技创新和成果转化机制，争取在环保产业、新材料产业发展上取得突破。构建各类教育协调发展的新格局，提高基础教育办学水平，重点发展高等教育、职业教育和优质高中教育。鼓励发展民办教育，扩大教育资源总量。全面完成农村中小学布局调整和危房改造。到２００７年，高等教育毛入学率达到１８％，基本普及高中阶段教育。高起点制定规划，动员各方面力量，积极发展信息产业，大力推进经济和社会信息化，打造“数字马鞍山”。

——体制机制实现新变革。以更大的决心和力度攻坚，突破一切影响发展的体制性障碍，构建与世贸组织和市场经济规则相适应、充满活力的体制基础。

深化产权制度改革。全面完成国有集体中小企业改制。推动大型企业产权多元化改革取得决定??。按照中央部署，完善国有资产管理体制，建立权利、义务、责任相统一，管资产与管人、管事相结合的资产运营体系。强力推进国有经济战略性调整，推动经营性资产向优势企业、优秀经营者集中。

进一步健全社会保障体系。巩固和完善养老、失业、医疗保险，实施工伤保险，启动生育保险，推进农村社保体系建设，建立对低收入者的救助制度，充分发挥社会保障制度“安全网”和“减震器”的功能作用。

毫不动摇地加快发展个体私营经济。培育、转制、引进并举，扫除障碍，放宽领域，放开手脚，调动一切积极因素，把广大人民群众创业创新的热情、智慧和力量激发出来，大办民有、民营、民享的民营经济，推动个体私营经济以快于全市经济的增速发展，力争到２００７年，个体私营经济在国内生产总值中所占比重增加到３０％。

进一步转变政府职能。继续深化行政管理体制改革，全面推进事业单位改革。继续实施城市管理重心下移，发挥区街功能。深化行政审批制度改革，不断深入地清理和减少行政审批及各类行政性收费，在开发区等有条件的地方试行“无费区”。完善政务公开和政风评议，变行政型管理为服务型管理。引入竞争机制，强化培训和管理，努力建设一支素质优良、作风过硬、能够担当重任的公务员队伍。

——城市化迈上新台阶。坚持城市“东扩南进”总体战略，注重城北与南京的衔接，全面加强城乡规划建设，大力推进城市现代化、城乡一体化和生态建设，加速城市化进程。到２００７年基本形成大城区框架，实行组团式发展，建成区面积力争增加到７０平方公里，初步形成布局合理、功能配套的城镇体系，力争城市化率达５８％。

高水准建设城乡基础设施。配合建成马芜高速公路，实施乡镇“半小时上高速”工程，抓紧开展南京轻轨线延伸至我市的项目前期工作，加速构建接轨长江三角洲的路网体系。实施城市主干道向东、向南延伸工程。南延湖东路，与湖西路、江东大道共同跨过采石河向南推进，与当涂县城对接。湖北路、湖南路跨越东环路、慈湖河向东延伸，建设慈湖河路、秀山路，拓展“东扩”构架。按计划建设沿江大道，为滨江发展创造条件。配合宁芜铁路复线改造，同步实施市内主干道平交改立交工程。同时，高水平改造一批市内主次干道，大幅度提高市内交通水平。加强公用设施建设，进一步提升水、电、气、通信等配套功能。实行高标准、大面积的区域性整体开发，加快建设新区。推进综合开发，继续实施旧城改造，重点解决城区居民危房问题，五年内基本完成市区成片危旧房和“城市中的村庄”改造。实施江河治理、防洪、排涝、水库除险加固、抗旱综合治理、水土保持等六大工程，增强城乡防洪保安能力。加大对农村的基础设施投入，改善农民生产和生活条件。

因势利导发展小城镇。编制城镇体系规划，形成中心城区—卫星城—重点镇—中心村的合理布局。坚持以产业支撑、特色兴镇，开辟市场化筹资渠道加快建设，完善小城镇功能。到２００７年，当涂县城面积达到１５平方公里，人口达到１５万；博望等城镇的集聚、辐射能力明显增强。

强化生态环境建设。完善城市水系的规划和建设，综合治理采石河、慈湖河，建成集防洪效益、生态效益于一体的连续、开放的绿地景观带；新辟道路同步实施高品位的环境景观建设，构建与现代化城市相匹配的新的园林体系框架。全面整治雨山湖，推进佳山、雨山景观改造，建设园林城市核心精品。高度重视农村生态保护，保持青山秀水的田园风光。实施城乡绿化一体化，建设“绿色马鞍山”。到２００７年，城市人均公共绿地面积达１０平方米，绿化覆盖率达４５％，全市森林覆盖率达１６??气、噪声污染治理和固体废弃物的处理，全面改善城乡环境质量，促进可持续发展。

——人民生活达到新水平。推进发展和变革，不断满足全市人民日益增长的物质文化需求。加快发展文化艺术和文化产业，进一步提高广播电视服务水平。分期逐步建设大剧院、博物馆、新图书馆、新科技馆。努力建设人人学习、终身学习的“学习型城市”。大力发展卫生、体育事业，提高市民的健康素质。继续稳定低生育水平，推动人口与计划生育工作持续健康发展。大力促进就业再就业，五年新增就业岗位８．５万个以上。积极营造有利于创业创新的氛围，保护一切合法收入，放手让一切劳动、知识、技术、管理和资本的活力竞相迸发，让一切创造财富的源泉充分涌流，让全市人民更快地富裕起来。到２００７年，力争全市人均国内生产总值达到３０００美元，城镇居民人均可支配收入超１万元，农民人均纯收入达４０００元，中等收入家庭比重不断提高，城乡居民家庭财产明显增加，过上更加宽裕的生活。坚持不懈地加强精神文明和政治文明建设，深入推进文明城市创建和依法治市，大力塑造创新、开放的城市文化，促进人的全面发展。

三、２００３年的重点工作

２００３年是新一届政府的开局之年，也是实施“十五”计划的关键一年。今年政府工作的总体要求是：以邓小平理论、“三个代表”重要思想为指导，深入贯彻党的十六大精神，以全面建设小康社会统揽工作全局，调动一切积极因素，推动改革开放、经济建设和各项社会事业取得明显进展，人民生活继续改善，为实现今后五年的奋斗目标、在加快皖江开发开放中率先突破迈出坚实的一步。全市经济和社会发展的主要预期目标为：国内生产总值增长１２％，财政收入基本同步增长，固定资产投资增长５０％以上，外贸进出口增长１０％，实际利用外资５０００万美元、内资１０亿元以上，城镇居民人均可支配收入和农民人均纯收入分别增长８％和５％，城镇登记失业率控制在４．５％以内，人口出生率控制在省下达的指标之内。为此，必须着力抓好以下九个方面的工作：

（一）不失时机地推动大开放迈出更大步伐。开发区利用外来投资要有明显增加，并有千万美元以上和亿元人民币以上投资的大项目，在全市招商引资中发挥龙头作用。基础设施建设要取得更大进展，配套完善西区的公用设施，基本建成东区３．６平方公里路网主体工程，启动南区起步区建设。加快慈湖工业园建设，年内２平方公里起步区基础设施建设和招商引资都要取得阶段性成效。各级领导带头，全面推行招商引资目标责任制，并奖惩兑现，进一步调动各方面积极性，面向海内外，多方式、多渠道、全方位开展招商引资。引导和督促大企业、大单位在利用外来投资上取得突破性进展。大力引进出口加工型企业，培育外贸增长点。加强指导与服务，落实鼓励出口政策，引导更多的企业开拓国际市场。整合外经资源，形成整体优势，促进工程技术承包、劳务输出等业务有较大增长。加强口岸建设，争取马鞍山港对外籍轮开放，进一步改善外贸条件。注重引进沿海地区特别是长江三角洲地区优质要素，支持本市企业积极参与区域产业分工合作。加大政府推动力度，充分发挥市场机制的作用，与南京及芜湖、铜陵的合作取得实质性进展，在“双赢”中实现共同发展。

（二）全力推进企业改革攻坚取得整体突破。加快企业产权多元化改革，促使大型企业通过经营者持股、引资重组、产权转让等多种形式进行改制，年内改制面达到８０％以上。支持上市公司开展资产重组和资本运营，积极培育高质量的上市后备队伍。对中小型企业，继续实施多种形式的改制，年内完成改革任务。健全改革推进机制，分层次、分系统组织队伍，精心筹划，集中力量抓好大型国有企业的改革，指导帮助县区搞好中小企业改革，支持帮助省部属企业改革。加大养老和失业保险金扩大征缴面、提高征缴率的力度，调整财政支出结构，增加社保资金支出，努力实现基金收支平衡。进一步完善城镇职工医疗保险政策，同步推进医疗机构和药品流通体制改革，尤其要引进高水平的医疗机构，加大药品招标采购力度，促进药品零售市场公平竞争，有效降低药品价格，提高服务质量。制定并实施工伤保险办法。支持国有商业银行实施综合改革，推动地方商业银行吸纳民间资本、利用外资进行重组扩股，组建农村信用联社，增强金融服务和发展能力。

（三）着力培育工业经济新的增长点。实施项目推进战略，进一步调动多元投资主体积极性，加快在建重点工业项目进度，年内新开工建设２０个重点工业项目，争取今年完成工业投资４０亿元以上，在加大工业投入强度上取得新突破，培育一批新的增长??马钢加快建设以薄板工程为重点的一批技改项目，并在发展非钢产业上取得明显进展，提高综合竞争力。积极扶持骨干企业实行高起点技术改造，加速形成规模经济。星马５０００辆专用汽车等重点技改项目建成投产，山鹰３０万吨纸板、金星６０００吨钛白粉等项目开工建设。创造条件促进外来投资企业、民营企业加快项目建设进度，花大气力引进加工制造业项目，尤其要鼓励和支持国内外知名企业在我市建设产业基地，加速产业结构升级。依托骨干企业，抓好一批重大项目前期工作。加强中小企业社会化服务和技术创新体系建设，推动中小企业逐步融入国内外产业分工体系，大力发展科技型、配套型、特色型产业。引导和促进企业创立名牌，建立科学管理制度，提高经济效益。今年争取工业保持２０％以上的增长速度，规模以上工业企业新增２０户左右，工业对全市经济增长的贡献份额继续加大。强化工业意识，坚定不移地走工业立市、强市之路，加快新型工业化进程。

（四）进一步推动个体私营经济提升发展水平。拓宽发展领域，鼓励有实力的私营企业采取多种形式参与国有集体企业的改制改组。支持发展势头好的私营企业依托品牌扩张，壮大规模和实力。引导有条件的私营企业进行公司制改革，提高资本运营能力。抓住沿海地区私营企业寻求扩张的机遇，大力引进优势私营企业来我市发展。扎实开展个私经济服务年活动，积极探索工商注册登记改革，完善企业检查、收费登记制度。健全投资信息发布机制，引导民间资本投向。支持组建中小企业融资担保机构，解决私营企业融资难问题。对纳税多、创汇多、创造就业岗位多的私营企业，实行奖励。对拥有自主知识产权的私营企业，在科技计划经费上予以重点倾斜。坚决制止乱收费、乱摊派、乱检查，保护个私企业的合法权益。全年个私经济增加值在国内生产总值中的比重达２３％以上，纳税额超１．５亿元。

（五）大力促使县区经济快速崛起。壮大县区经济是马鞍山实现跨越式发展的必然选择，市政府将进一步加强对县区经济发展的指导和扶持，各县区必须树立雄心壮志，聚精会神发展生产力，专心致志把经济搞上去，加速建设经济强县强区。要以特色园区为载体加速工业化进程。当涂县年内完成工业园２平方公里起步区基础设施配套，加快在建项目进度，尽快出效益、出形象，争取挤进省重点工业园区行列。支持各区全面启动工业、旅游园起步区建设。加强乡镇工业小区的规划和建设，提高资源利用效率。各县区要把园区建设与中小企业的搬迁改造和招商引资结合起来，让园区成为拉动县区工业增长的主力。以龙头企业为抓手推进农业产业化。引进和培育并重，壮大龙头企业群体，力争年内有２户企业销售收入超亿元，５户企业超５０００万元。推进龙头企业生产基地建设，启动实施大公圩农业绿色开发园区项目。大力发展农产品营销等中介服务组织，改建、扩建一批农产品批发市场，畅通农产品流通“绿色通道”。加快农产品质量标准和检验检测体系建设，建成农产品安全质量监测中心，抓好“放心食品工程”。集中抓好重点镇的基础设施建设，完善配套功能。因地制宜培育特色产业，发挥小城镇聚集和辐射作用。多渠道促进农民增收。加大对农业的支持，提高农业综合效益。积极开展多种形式的农村就业培训，提高农民的综合素质和择业能力。增强各级劳务输出服务机构的功能，及时传导劳务信息，提高劳务输出组织化程度。巩固发展农村税费改革成果，加强对涉农收费的管理，减轻农民负担。

（六）全面加强城乡规划建设。完成新一轮城市总体规划的修编，制定集贸市场和商业网点规划，加快其它专项规划和控制性详规编制，更好地发挥规划导向作用。积极争取国债项目，继续搞好经营城市工作，多渠道筹措建设资金，加大对基础设施建设的投入。建成马濮旅游公路和当高大桥，改建围屏至乌溪公路，进一步改善交通条件。新建湖东南路、中心大道、印山路和花园路，贯通九华路，拓宽改造红旗中路南段，启动建设市中心广场，带动南部新区开发。开工建设湖南东路，新建林里路，改造林里桥，促进城市“东扩”。对主干道交叉口拥挤状况进行综合整治，提高通行能力。实施老旧小区和背街小巷综合整治二期工程，进一步改观老城区和老居住区面貌。完成天然气利用一期工程，推进四水厂、第二污水处理厂建设，动工建设江东２２０千伏输变电工程等电力设施，提升城市承载能力。积极推进房地产开发建设，建成一批不同档次、有一定规模的住宅小区。实施小型水库除险加固工程，做好长江马鞍山河段整治二期工程前期工作。启动采石河整治工程，开展整治慈湖河前期工作。加快锁溪河防洪工程建设，完成碧溪河综合整治、雨田路排水工程建设和雨山河１号排涝泵站改建，进一步增强城市防洪排涝能力。抓好园林绿化，建成朱然公园，筹建花果山公园。对部分道路进行绿化和游园改造，实施亮化美化。整顿规范路街巷标志和门牌，推进地名标准化建设。加快矿山环境整治和土地复垦，逐步恢复自然生态。完成退耕还林２．２万亩，提高森林覆盖率。继续加强城市环境综合治理，严格控制新的污染源的产生，开展生活垃圾分类收集试点，实施雨山湖截污清淤工程。深化城建体制改革，积极推进有稳定收益的城市基础设施的市场化经营，通过改制重组推动公用管理事业单位走向市场。强化对土地一级市场的管理，大力推进国有土地资本运营。完善土地征迁办法，加大土地征迁力度，保证重点建设用地的需要。完善和规范相对集中行政处罚权试点工作，加强城市管理，强化违法建设拆除工作，突出抓好流动摊点等重难点问题的整治。

（七）深入实施科教兴市战略。鼓励和促进重点骨干企业密切与高等院校、科研机构的合作。支持矿院启动建设国家级金属矿山固体废物处理与处置工程技术研究中心。鼓励科技人员、社会力量领办和创办民营科技企业。完善和发挥高新技术创业服务中心功能，以“８６３”新材料项目为重点，加强产学研合作，促进科研成果转化和高新技术产业化。以电子政务建设为突破口，启动实施信息化发展规划。继续把教育摆在优先发展的地位，巩固发展“两基”成果，提高基础教育质量。加强当涂县中小学危房改造和布局调整。建成二中新校区。争取当涂一中等学校跨入省示范高中行列。支持安工大完善设施，启动软件职业技术学院建设，争取设立博士学位授予点。年内建成网络大学一期工程。加快筹建高等师范专科学校，建成新校区。力争启动建设安徽工贸技师学院。培育多元化教育投资主体，鼓励和扶持民办教育发展。高度重视人才工作，树立“不求所有、但求所用”的新观念，实施人才柔性流动政策，积极引进高层次、紧缺专业人才。有计划地选派年轻人才到国内外高等院校深造，继续做好有突出贡献的中青年专家、学术带头人的选拔培养工作。建立科学的人才评价和使用机制，营造人才成长和创业的良好环境。

（八）努力扩大就业和改善人民生活。认真落实积极的就业和再就业政策，加快城乡就业服务体系建设，深入开展“创岗就业”社会行动，建立社区就业平台，积极发展劳动密集型企业，吸纳劳动者就业。强化下岗失业人员技能培训，鼓励和扶持他们自谋职业、自主创业和组织起来就业，对困难群体实行再就业援助，多渠道、多形式促进就业和再就业。加强和规范城市“低保”工作，建立因病及其他突发性因素致困救助机制，保障特困家庭的基本生活。深化住房制度改革，建立廉租房制度，完善住房供应体系，发展和规范物业管理。积极推进分配制度改革，切实保障各种合法收入，同时依法调节收入差距，逐步实现合理公平分配。今年，实施央视７套节目进村入户，新建一批市场、停车场和生活垃圾中转站，实施成片危旧房和“城市中的村庄”改造一期工程，启动帮助青年创业计划和就业信息系统建设等与群众利益密切相关的实事项目，当年实施、当年受益。

（九）促进精神文明建设和社会各项事业协调发展。深化文明行业、文明单位、文明社区、文明村镇、文明家庭创建工作，巩固发展创建成果。继续加强公民道德建设，深入开展多层次的道德教育与实践活动，提升全市人民文明素质。适应全面建设小康社会的要求，大力发展文化、体育、广播电视事业。深化城市医疗卫生体制改革，完善城市医疗服务体系。增强社会健康意识，切实加强血吸虫病、性病、艾滋病等传染病的防治工作。扎实推进农村改水改厕，健全农村卫生网络，加快建立新型农村医疗制度，提高全民卫生健康水平。加强社区建设和管理，建立和完善社区人口与计生管理服务机制，认真搞好农村和流动人口的计生管理服务。深化户籍管理制度改革，引导人口有序进入城镇。发展老龄事业，重视妇女儿童工作，维护残疾人合法权益，做好对台、侨务和民族宗教工作。全面启动市、县区志书编修工作，加强统计、档案等基础性工作。落实领导责任制及领导责任追究制，进一步做好信访工作。加大社会治安综合治理力度，严厉打击各类违法犯罪活动。全面贯彻《安全生产法》，强化安全生产责任，坚决杜绝重特大事故发生。进一步深化城市民兵工作改革，不断完善国防动员体制，推动“双拥”工作深入开展。

四、加强和改进政府自身建设

今后一个时期，要实现马鞍山跨越式发展，必须以改革的精神全面推进政府自身建设，不断提高施政水平。

大兴学习之风，建设学习型政府。全体公务员特别是各级领导干部要积极顺应时代发展的潮流，牢固确立学习为本、终身学习的理念，不断更新知识、转变观念，使自身素质与时代要求相适应，使知识和本领与承担的工作任务相适应。当前，要把认真学习十六大精神作为首要的政治任务，深入领会和努力实践“三个代表”重要思想。要把学习与调研结合起来，坚决克服坐而论道、空发议论、做表面文章的不良学风。要坚持学习与运用相统一，以经济建设为中心，紧密联系实际，着眼于知识的运用，着眼于对现实问题的思考，着眼于新的实践和发展，切实解决本地区、本单位存在的突出问题。要鼓励和支持公务员开展多种形式的学习，形成比学习、比奉献的浓厚氛围。

加快转变职能，建设服务型政府。以深入整治经济发展环境为重点，推动政府职能的加快转变。继续削减审批事项和收费项目，对保留的审批事项实行相对集中办理，并建立行政审批责任制度，追究违法审批和失管责任。完善行政服务中心的运作机制和服务功能，将其建设成为运转灵活、协调高效的为投资者、为广大人民群众办事服务的“窗口”。实施事业单位改革，??全投诉受理网络，及时处理投资者、企业和群众的投诉，保护他们的合法权益。坚持不懈地开展整顿和规范市场经济秩序工作，集中整治与群众切身利益相关、群众关心、关系全局的问题，努力营造公平、公正、公开的市场环境。

坚持依法行政，建设法治型政府。加快推进政府工作法制化。严格执行规范化、程序化的决策制度，积极创新法律政策实施机制，统筹整合现有各项监督制度和监督力量，加强行政执法检查，切实解决行政管理中的不作为问题。各级政府要认真执行人大决议和决定，自觉接受人大及其常委会的法律监督和工作监督，加强与人民政协的协商，接受人民政协的民主监督，认真办理人大代表议案、建议和政协提案。广泛听取各民主党派、工商联以及社会各界的意见，发挥工会、共青团、妇联等人民团体的桥梁纽带作用。完善市长与市人大代表、政协委员约见制度，发挥市长公开电话作用，拓宽民主监督渠道。

切实改进作风，建设勤政廉洁型政府。全体公务员特别是各级领导干部要强化宗旨意识，坚持走群众路线，尊重群众的首创精神，汲取群众的智慧和力量。力戒形式主义、官僚主义，不务虚名，不做表面文章，雷厉风行抓落实。完善目标管理，实行重点工作跟踪督查，建立有效的工作落实机制。加大考核力度，发挥奖惩的导向作用，营造能干事、能干成事的良好氛围。加强行政监察和审计监督，强化对权力部门和岗位的监督管理。弘扬艰苦奋斗的传统，抓好增收节支工作，狠刹奢侈浪费歪风。大力推行国库集中支付、部门预算管理、会计委派制度，完善政府采购、招投标制度，建立政府投资项目集中统一管理制度，提高行政效率，从源头上预防和治理腐败。

各位代表，勤劳智慧的马鞍山人民在过去的岁月里创造了历史的辉煌。在党的十六大精神的指引和鼓舞下，经过市场经济大潮洗礼的马鞍山，已踏上追赶奋进、跨越发展的新征程。升腾的希望感召着我们，宏伟的目标激励着我们，我们的任务光荣而艰巨。让我们高举邓小平理论伟大旗帜，全面贯彻“三个代表”重要思想，在省委省政府和市委领导下，团结一致，真抓实干，开拓前进，谱写富民强市的新篇章，创造马鞍山人民的幸福生活和美好未来！
